# A Scientometric Review of Urban Disaster Resilience Research

**DOI:** 10.3390/ijerph18073677

**Published:** 2021-04-01

**Authors:** Hui Xu, Yang Li, Yongtao Tan, Ninghui Deng

**Affiliations:** 1 School of Economics and Management, Chongqing University of Posts and Telecommunications, Chongqing 400065, China; s180701025@stu.cqupt.edu.cn (Y.L.); dengningh@163.com (N.D.); 2 School of Engineering, RMIT University, Melbourne, VIC 3001, Australia; yongtao.tan@rmit.edu.au

**Keywords:** urban disaster resilience, sustainable development, vulnerability, resilience assessment, keyword statistics

## Abstract

Natural disasters and human-made disasters are threatening urban areas globally. The resilience capacity of the urban system plays an important role in disaster risk response and recovery. Strengthening urban disaster resilience is also fundamental to ensuring sustainable development. Various practices and research for enhancing urban disaster resilience have been carried out worldwide but are yet to be reviewed. Accordingly, this paper gives a scientometric review of urban disaster resilience research by using CiteSpace. The time span (January 2001–January 2021) was selected and divided into three phases based on the number of publications. In addition, according to keyword statistics and clustering results, the collected articles are grouped into four hotspot topics: disaster risk reduction, specific disaster resilience research, resilience assessment, and combination research. The results show that most of the existing research is in the first two categories, and articles in the second and fourth categories both show a high growth rate and could be further research directions. The review indicates that urban disaster resilience is essential for a city’s sustainable development. Moreover, the findings provide scholars a full picture of the existing urban disaster resilience research which can help them identify promising research directions. The findings can also help urban government officials and policymakers review current urban disaster management strategies and make further improvements.

## 1. Introduction

Urban disasters, including natural hazards, equipment accidents, public health events, and terrorist attacks are increasing in recent years exponentially, resulting in escalating economic and human losses, and threatening urban sustainable development [1,2]. On 22 January 2021, Aon plc released a statistical report, “Weather, Climate & Catastrophe Insight 2020 Annual Report”. According to the report, the global direct economic losses and damages from natural disasters in 2020 were estimated at USD268 billion. Although much lower than the peak loss years of 2011 (USD557 billion) and 2017 (USD485 billion), it was above the average (USD244 billion) and median (USD246 billion) of the 21st century. Meanwhile, approximately 8100 people lost their lives due to natural catastrophe events in 2020 [3]. These economic losses and fatalities stemmed from multi-hazard disasters, including seasonal floods, hurricane Laura, cyclone Amphan, etc. Moreover, at the beginning of 2020, COVID-19 swept across the world and became the deadliest pandemic on the planet since the 1918 influenza pandemic. The World Health Organization estimated that more than 10% of the world’s population may have been infected with the new coronavirus [3]. Despite the scientific and technological developments, it is still not possible to accurately predict and prevent the occurrence of some disasters [4].

According to the statistics, the number of disasters worldwide has almost quadrupled during the past 30 years [5]. Urban disasters present unique characteristics in the sense that cities are in an environment that absorbs large volumes of populations and facilities. Consequently, urban authorities and actors are facing challenges of establishing strategies for various kinds of disaster risk reduction and adaptation and further promoting the sustainable development of cities.

The concept of “resilience” represents a system’s long-term ability in coping with disturbance and maintaining development, which is a defining property of complex systems [6,7]. This concept originally derives from the Latin word “resilio”, which means to “bounce back” [8]. In 1973, the concept was first formally introduced by Holling in the field of ecology [9]. Then, it was used in the aspects of psychiatry and psychology, which were reflected in the work of Norman Garmezy, Ruth Smith, and Emmy Werner [10,11,12]. After this, the utilization of the concept in the published works presented a clear upwards trend [13]. Especially in 2001, the interest in resilience was greatly triggered by the World Trade Center attack in the United States [14]. Gradually, this concept was used in the fields of ecology, psychology, economics, engineering, and urban development, etc. [8,15].

The common use of the term resilience means the capacity of a system to reorganize and return to a normal condition after a sudden shock [8]. Resilience capacity includes absorptive capacity, adaptive capacity, and restorative capacity [16,17,18]. Absorptive capacity is the ability of a system to absorb shocks from a disruptive event and minimize the destructive consequences [19]. Adaptive capacity is the ability of a system to make intentional incremental adjustments to adapt itself to a disruption [20]. Restorative capacity is the ability of a system to recover from a disruption. “Resilience” has been increasingly concentrated in the risk response literature [13]. The previous strategies emphasizing the preparedness planning for disruptive events may not be sufficient due to the impossibility that strategies would be able to cope with all types of disasters. “Resilience” supplies and focuses on a timely response and recovery from a sudden disruption [19]. The resilience of urban areas in the face of various disruptive disasters is becoming an increasingly important topic among scholars and governors in recent years [21,22].

Further, the concept of “sustainable development” has also been emphasized in the urban resilience studies by governments and scholars, which aim to make cities more “disaster resilient” and “sustainable” in the fields of urban disaster prevention and mitigation [20,23,24]. The concepts “disaster resilience” and “sustainable” are related to each other. On 1 January 2016, the 17 Sustainable Development Goals of “the 2030 Agenda for Sustainable Development”, adopted by world leaders at the historic UN Summit in September 2015, officially came into force. The 11th goal is to “make cities inclusive, safe, resilient and sustainable”, including that by 2020, substantially increasing the number of cities and human settlements that adopt and implement integrated policies and plans towards resilience to disasters, and in line with the Sendai Framework for Disaster Risk Reduction 2015–2030, holistic disaster risk management at all levels [25]. Moreover, the close relationship between these two concepts has also been emphasized in many international academic studies. The sustainability of the urban system was considered in disaster resilience assessments, as both disaster resilience and sustainability could reduce risks and supply resources to post-disaster recovery [26,27]. Disaster resilience was proposed as a foundation for sustainability in social sustainability-oriented development and resilience planning [2,28,29]. Therefore, urban disaster resilience presents a high necessity in the promotion of urban sustainability and is conducive to the sustainable development of cities.

However, a systematic review of urban disaster resilience research is yet to be presented. Accordingly, this paper provides a scientometric review of urban disaster resilience research based on keyword statistics. The existing studies (from January 2001 to January 2021) in the field of urban disaster resilience are divided into three phases based on the publication numbers, including the initial phase, the gradual development phase, and the rapid expansion phase. Further, according to the keyword statistics and clustering results, the collected articles are grouped into four hotspot topics: (1) disaster risk reduction, (2) specific disaster resilience research, (3) resilience assessment, and (4) combination research (combining the concepts of sustainability and vulnerability). The review of the literature is conducted from four categories. The discussion section summarizes the findings and gives some recommendations for future research.

## 2. Research Methodology

At present, global scientific databases include Web of Science, Scopus, PubMed, Google Scholar, and Derwent, etc. Among the databases, the Web of Science Core Collection (WoSCC) database covers almost all the most comprehensive and authoritative scientific literature worldwide. Numerous leading journals and detailed information about publications around the world are available from the WoSCC, which remains the most widely used database in the field of natural science [30,31]. In addition, the WoSCC could meet the special requirements of CiteSpace for data structure and content. Therefore, the WoSCC has been used as the data source for bibliometric investigations in this research. The reviewed articles in the field of urban disaster resilience are retrieved from the WoSCC (updated to 22 January 2021), and all articles are indexed by “Science Citation Index Expanded (SCI-EXPANDED) (since 1990)”, or “Social Sciences Citation Index(SSCI)(since 2016)”, or “Conference Proceedings Citation Index-Science (CPCI-S) (since 2011)”, or “Conference Proceedings Citation Index-Social Science & Humanities (CPCI-SSH) (since 2011)”, or “Current Chemical Reactions (CCR-EXPANDED) (since 1985)”, or “Index Chemicus (IC) (since 1993)” [8]. The keywords in the literature search are the combination of three key terms, “urban”, “disaster”, and “resilience”. For every key term, several words with similar meanings are also used in the literature search. As for “urban”, the words “urbanization”, “urbanism”, “urbanize/urbanized”, “city”, and “cities” are all considered. Moreover, “community” or “communities” were used as alternative key terms to urban, because community is the main space in urban areas that many resilience studies focus on [32,33,34,35]. In the consideration of “disaster”, the terms “accident”, “incident”, “calamity”, “catastrophe”, “emergency”, “risk”, “hazard”, “safe/safety”, and “secure/security” and the plural forms are all considered. In addition, “resiliency” and “resilient” were used as alternatives to “resilience”. To increase the study accuracy, the “advanced search” and “operators (AND, OR)” were used for searching. The key terms and search strategy are as follows: TI = (((“urban*”) OR (“city”) OR (“communit*”) OR (“cities”)) AND ((“disaster*”) OR (“disastrous”) OR (“accident*”) OR (“incident*”) OR (“calamit*”) OR (“catastroph*”) OR (“emergen*”) OR (“hazard*”) OR (“risk*”) OR (“safe*”) OR (“secur*”)) AND ((“resilient”) OR (“resilience”) OR (“resiliency”))). Along with the searching rules, the timespan for searching articles was set from 1991 to 2021. A total of 488 items of literature were retrieved (accessed on 24 January 2021), which include 336 journal articles, 86 proceedings papers, 21 reviews, 20 meeting abstracts, 19 editorial materials, 4 book reviews, 1 letter, and 1 news item. Only the literature that meets the following eligibility criteria would be screened in the review.

### 2.1. Eligibility Criteria

#### 2.1.1. Inclusion Criteria

Topic: On the basis that the title of an article satisfies the above search strategy, the research content must conform to the disaster resilience research in urban areas.

Publication year: Retrieved literatures that are between January 2001 to January 2021. It is worth noting that there was no literature on urban disaster resilience before 2001. The main reason is that the term resilience was mainly applied to social-ecosystems in the 1990s, little attention was paid to the studies related to urban disaster resilience from 1991 to 2000.

Language: Only articles written in English were eligible for inclusion.

Publication status: Only international peer-reviewed journal articles were considered for further analysis.

#### 2.1.2. Exclusion Criteria

Articles were excluded for (1) research in the field of psychology and (2) research in the field of psychiatry. Through reviewing the titles and abstracts of journal articles, the articles in the fields of psychology and psychiatry were excluded. In addition, publications such as proceedings paper, review, book review, abstract, editorial material, letter, and news, etc., were not considered in this review. Due to length limitations, conference papers from annual meetings lack important information needed to systematize (such as statistical and methodological crucial information, concrete analysis procedures), and the methods are often introduced into journal articles [36,37]. Moreover, other types of publications are almost not involved in research hotspots or research frontiers of significance due to the publication length or lack of key information.

### 2.2. Data Analysis

CiteSpace, a java application for analyzing and visualizing literature through the network [38], was adopted in this study to analyze the potential knowledge contained in keywords. The primary aim was to analyze emerging trends and predict future directions in a knowledge domain [39]. The analysis for the literature was based on keyword statistics. As the core vocabulary and phrase of an article, keywords are a highly concise summary of the article’s theme. Keywords in an article can provide relevant information, such as research object, method, and research hotspots. Therefore, according to the above determined literatures, the time threshold was set on CiteSpace from January 2001 to January 2021, and one year was selected as the time slice. To remove redundant information and to ensure the clarity of the view, data from the top 50 levels of most frequently occurring nodes in each time slice were screened, and the combination strategy for pruning contained “Pathfinder” and “Pruning sliced networks”. After completing the above configuration, the “Author Keywords (DE)” and “Keyword” were selected successfully as the term source and node type, respectively.

**Figure 1:**
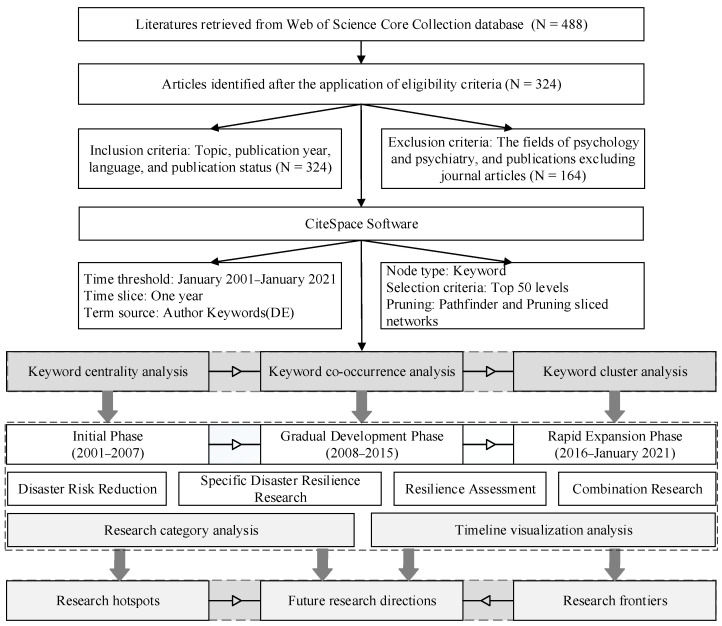
Outline of research design.

The whole process from literature retrieval to data analysis was conducted in three steps, which are (1) the identification of literatures from the WoSCC database, (2) the application of eligibility criteria, and (3) the configuration of CiteSpace software. Subsequently, a series of statistics and analyses based on keywords has been conducted. The outline of the research design is shown in Figure 1.

### 2.3. Statistics for the Number of Publications

**Figure 2:**
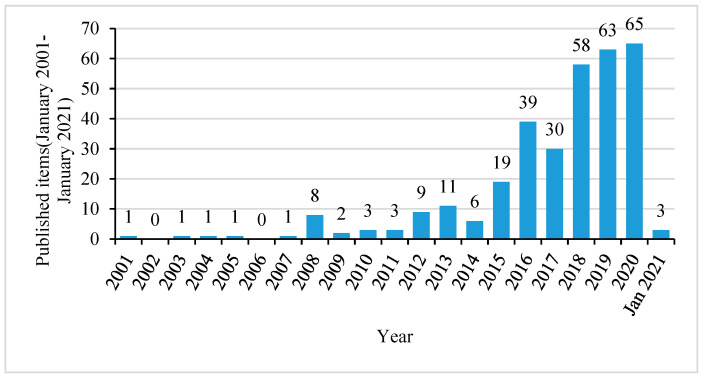
Annual distribution of the articles on urban disaster resilience.

The distribution of articles by year is shown in Figure 2. According to the number of published articles, the research on urban disaster resilience can be divided into three phases, which are Phase I–initial phase (2001–2007), Phase II–gradual development phase (2008–2015), and Phase III–rapid expansion phase (2016–January 2021) (Table A1). In Phase I, there are only five published articles. In Phase II, the number of published articles each year is less than 20, and in total 61 articles were published. In Phase III, the research on urban disaster resilience shows an obvious increasing trend, and more articles were published every year than in the previous stage, with a total of 258 articles published.

### 2.4. Statistics for the Publication Journal

According to the number of publications automatically sorted by WoSCC, as shown in Table 1, the top 10 journal sources were determined, all of which have recorded five or more published articles in the field of urban disaster resilience. It can be seen that 37 articles referring to urban disaster resilience are published in the *International Journal of Disaster Risk Reduction*, 22 in *Natural Hazards*, and 11 in the *International Journal of Environmental Research and Public Health* and *Sustainability*, respectively. In total, 118 articles are published in the top 10 journals with focuses on disaster risk reduction, natural hazard mitigation, emergency management, and built environment resilience.

## 3. Results

### 3.1. Keyword Centrality Analysis

The betweenness centrality of the network is the ability of a node to occupy the shortest path between the other two nodes, which is a measure of the ability of a node as a “bridge” [40]. The higher the betweenness centrality is, the more nodes that this node occupies, and the more information among keywords that this node controls [41]. Keywords with centrality higher than or equal to 0.1 are more important than that below 0.1 [42]. According to Table 2, the centrality of the top 17 keywords is 0.1 or above. Among the top 10 keywords, “social ecological system” shows the characteristics of the urban system, “policy”, “challenge”, “hazard”, and “disaster” are the research subjects, and “preparedness”, “disaster risk reduction”, “reflection”, “perception”, and “risk reduction” show the resilience ability.

### 3.2. Keyword Frequency Statistics

#### 3.2.1. Keyword Expansion from Phase II to Phase III

**Figure 3:**
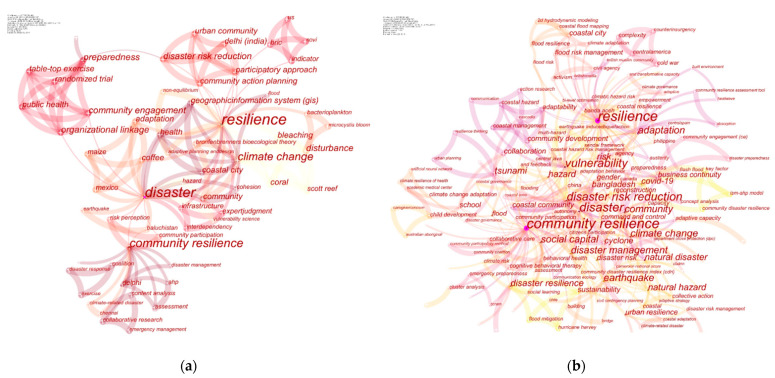
Keyword co-occurrence knowledge map of Phase II (**a**) and Phase III (**b**).

**Figure 4:**
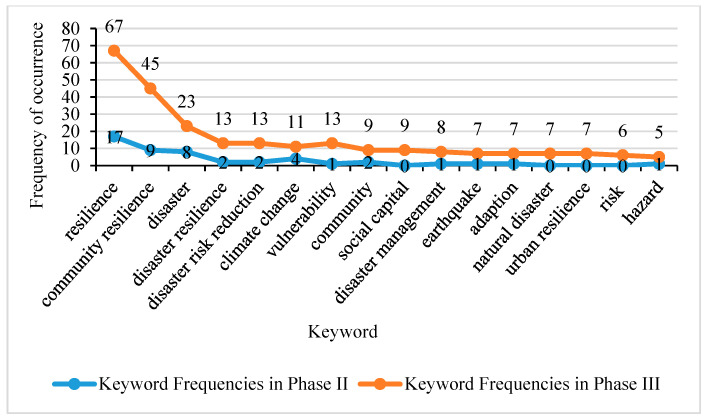
Frequency comparison of co-keywords in Phase II and Phase III.

A total of 319 articles were published in the last two phases (2008–January 2021). Keywords have expanded from Phase II to Phase III in number and frequency. The keyword co-occurrence knowledge map of Phase II and Phase III is shown in Figure 3. The statistics for the expansion trends of co-keywords from Phase II to Phase III are shown in Figure 4.

Moreover, keywords that first appeared in Phase III are shown in Table 3, which reflect emerging research directions and methods in recent five years.

#### 3.2.2. Keyword Frequency in the Three Phases

**Figure 5:**
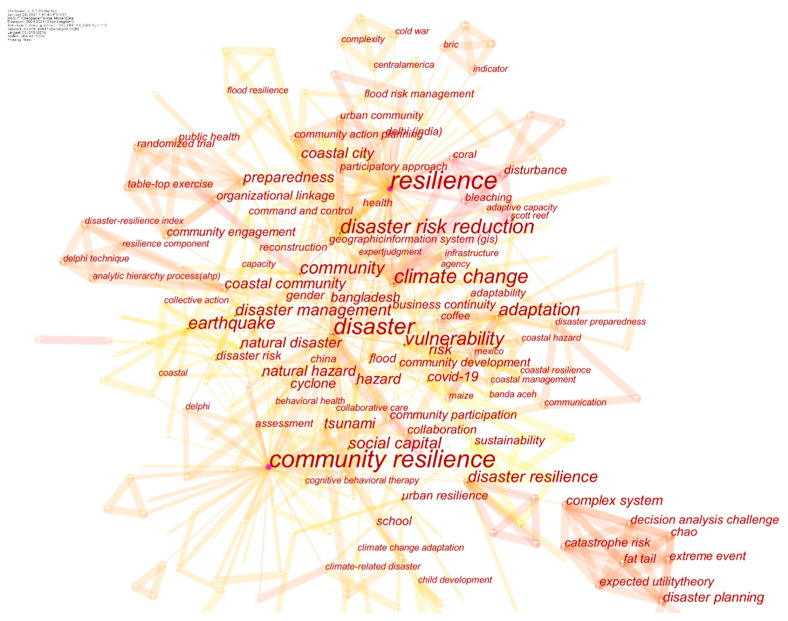
Keyword co-occurrence knowledge map of 324 articles.

The co-occurrence knowledge map of keywords in the 324 articles (articles in the three phases) is shown in Figure 5.

The co-occurrence analysis of keywords cited in the studies can reveal articles’ vital topics as well as the hotspot and frontier knowledge of the overall field. In the keyword co-occurrence knowledge map, a cross node represents a keyword. The larger the node, the higher the frequency of keywords, and the greater the attention the node presents. Accordingly, keywords with a frequency above 5 are shown in Table 4.

### 3.3. Keyword Cluster Analysis

The keyword co-occurrence network can be divided into many clusters of co-cited references. References are tightly connected within the same clusters, but loosely connected among different clusters. In the object analysis function panel of CiteSpace, the node type “Keyword” was selected successively as the clustering basis, and CiteSpace’s automatic clustering function was used to draw the scientific knowledge map. The size, the number of members in each cluster, is an essential prerequisite for cluster selection. Larger clusters with over 10 members tend to more representative than small clusters, which are likely to be grouped by the citation of a small number of publications. Moreover, the quality of a cluster is also reflected in terms of its silhouette value, namely, an indicator of its homogeneity or consistency. The silhouette score of a cluster tends to be close to 1, indicating that it is highly homogeneous. Table 5 lists eight major clusters considering the above conditions. The cluster label automatically selected by the log-likelihood ratio test method (LLR) mainly reflects a unique aspect of a cluster, and this method demonstrated by Professor Chaomei Chen is the best strategy for automatic cluster labeling [43]. Additionally, keywords with a frequency of not less than 5 were selected as the main keywords, which represent the research hotpots of each cluster.

In Table 5, these eight important clusters represent hotspot topics of urban disaster resilience research. Cluster #0 reflects the research topic “disaster risk reduction”. Clusters #1, #2, and #4 focus on the research of resilience, including resilience assessment research and specific disaster resilience research. Clusters #3, #5, and #6 display the study of specific disasters, such as climate change, tsunami, and earthquake, etc. The last #7 keyword cluster presents the combination research topic “vulnerability”. Thus, the four hotspot topics could be summarized as disaster risk reduction, specific disaster resilience research, resilience assessment, and combination research.

## 4. The Three Phases of Urban Disaster Resilience Research

**Figure 6:**
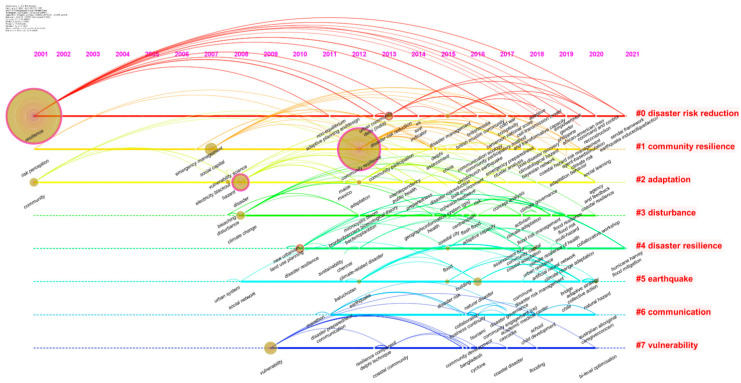
The timeline view of keywords in urban disaster resilience articles.

Based on the above statistics, the review for the urban disaster resilience research is conducted from the above four research categories. Attaching the time factor, timeline visualization will make newly emerged threads of research stand out so that they can be recognized more easily in each phase. Moreover, each cluster is displayed horizontally and advances over time from left to right. Consequently, Figure 6 shows a timeline visualization of keywords in urban disaster resilience research from 2001 to January 2021.

### 4.1. Phase I: Initial Phase (2001–2007)

#### 4.1.1. Timeline Visualization Analysis

During 2001–2007, a total of five articles were published, four of which were highly cited. As shown in Figure 6, the keywords that emerged at this stage indicate that new clues from journal articles have infiltrated into the field of “urban disaster resilience”. Among those keywords, three prominent circles depict the references to keywords and the persistence of the research topics, which are “resilience”, “community”, and “social capital”. Following the research process of resilience, it can be seen that along with a precursor, the concept of resilience had entered the urban realm as early as 1973. Since 2001, the theme of combining urban disaster and resilience at the community level has been explored, with the primary purpose of disaster response and loss reduction.

#### 4.1.2. Research Category Analysis

The initial phase of research is a creative beginning of the field, which is mainly summarized into three categories, including specific disaster resilience research, disaster risk reduction, and resilience assessment research. Specifically, in 2001, the published articles examined community resilience to volcanic hazards in predicting resilience to the social consequences [44]. Later, the article “Urban Hazard Mitigation: Creating Resilient Cities” was published in the *Natural Hazards Review* journal, with a citation frequency of 512. This article proposed a comprehensive urban hazard mitigation strategy to create resilient cities. The relationship between resilience and hazards was also discussed [45].

The third category includes three articles published in 2004, 2005, and 2007, all focusing on the disaster resilience of communities. The article published in 2004 focused on the development and application of resilience quantitative measures. It first proposed resilience measures, which consider expected losses in further disasters within a community scope, and the result showed that the resilience framework can be useful for guiding disaster mitigation efforts [46]. Moreover, the article published in 2005 was about resilient community establishment. Based on the background of the great Sumatra–Andaman Earthquake on 26 December 2004, and the resulting Indian Ocean tsunami, it aimed to explore measures in achieving more tsunami-resilient communities by addressing some key problems, such as poverty, education, emergency medical, and rescue services, etc. [47] The article published in 2007 discussed two types of emergency management, namely, the local-scale municipal government responsibilities and community-level initiatives. It demonstrated that these were interdependent but separate aspects of emergency management and proved that social capital resources were of critical importance to promote a community’s resilience to risks and hazards [48].

### 4.2. Phase II: Gradual Development Phase (2008–2015)

Articles in Phase II present two characteristics, including number growth and content expansion. A total of 61 articles were published in this phase. The four research topics mentioned previously, disaster risk reduction (26 articles), specific disaster resilience research (14 articles), resilience assessment (17 articles), and combination research (4 articles), were accordingly reviewed.

#### 4.2.1. Timeline Visualization Analysis

During 2008–2015, numerous new keywords emerged each year, accompanied by the expansion of articles in terms of volume and content. As displayed in Figure 6, the six keywords with a circle sharply stand out and reveal new developments in the field of urban disaster resilience since the 2008 scientometric study. The bigger the circle of a keyword, the more attention it receives, and the longer its research duration. Notably, four circles with purple rings indicate that these keywords have a high betweenness centrality, which acts as a bridge extending from earlier to more recent ideas. The above four keywords include “hazard”, “disaster resilience”, “community resilience”, and “disaster risk reduction”, which appeared in 2008, 2010, 2012, and 2013, respectively. Moreover, evidence from the number of articles in Figure 2 demonstrates that the combination of “resilience”, “community”, and “disaster” has become a hotspot research topic. Meanwhile, the practical value of the research lies in the pursuit of disaster risk reduction.

#### 4.2.2. Research Category Analysis

Based on the timeline visualization analysis, six keywords recognized as research hotspots are further analyzed according to references. The references with a high citation frequency can be directly identified in CiteSpace.

For disaster risk reduction, in recent years damages in the urban areas caused by a dramatic worldwide increase of natural and human-made disasters are staggering, with many people being at risk. Under this condition, some topics, including the key variables and their causal relations in disaster events [49,50], the impacts of disasters [51,52], the strategies to cope with disasters and mitigate risk influence [26,53,54], the reasons for weak coping [55,56], etc., have been explored in many studies. It is recognized that acceptance, self-reliance, spirituality, preparedness, resource availability, serving others, social support networks, and place-based social cohesion have positive impacts on disaster resilience [51,52]. Practical applications, such as mass sporting events, social media, settings across the space between household occupancy and business operations, community action planning, and public involvement, were considered as useful measures to enhance community disaster resilience [57,58,59,60,61]. In addition, some promotion strategies, such as the relationship between disaster recovery and the role of place and social capital, critical infrastructures disaster mitigation strategies, urban governance, integration of urban resilience in the earthquake reconstruction, urban planning, and design strategies were studied to enhance urban disaster resilience [50,62,63,64,65].

For specific disaster resilience research, research is conducted from angles of multiple specific disasters or problems. Natural disaster resilience is the main topic in this category, including establishing a model to improve natural disaster resilience at the community level [49], adapting ecological resilience into human community systems in response to natural disasters [66], evaluating how initial public wealth affects the post-natural disaster recovery and resilience for communities [67], employing Bronfenbrenner’s bio-ecological theory to model community resilience to natural disasters [68], developing an integrated framework to measure the resilience of urban systems against disasters [26], measuring the community resilience of an earthquake-prone area in Baluchistan [69], evaluating the promise and performance of New Urbanism to create a disaster-resilient community [56]. In addition, research focusing on specific disasters or problems, such as public health, flood risk, tsunami, climate change, public–private partnerships, and women’s empowerment, etc., was also conducted for urban resilience development and enhancement [68,69,70,71,72,73,74,75,76].

For resilience assessment research, a total of 17 articles are in this category. Resilience assessment is an important aspect in urban disaster resilience studies, which has been gradually attracting research attention in recent years. For instance, a novel risk assessment method for evaluating disaster resilience capacity of hillslope communities in debris flow and landslide was established using logistic regression analysis and Geographic Information System (GIS) technology [77], and further, the appropriated parameters for the assessment were explored in the subsequent work [78]. Moreover, a qualitative case-based research method was used in studying success factors for enhancing urban community disaster resilience [33]. A climate-related community disaster resilience assessment framework was established, and three dimensions (the physical dimension, social dimension, and economic dimension) were included [79]. In the context of earthquake hazards, a community resilience assessment index was developed, which contained social resilience, economic resilience, institutional resilience, and physical resilience [69].

For combination research, two important concepts, sustainability and vulnerability, are combined in the urban disaster resilience research. Four articles from top-ranked journals are included in this category, among which three are related to sustainability and one with vulnerability. The concepts of resilience and sustainability are extremely relevant in the sense that the consequences of natural disasters influence the social, environmental, and economic burden of cities. The urban infrastructure systems are widely acknowledged to be a lifeline of the urban system and play a vital role in urban resilience and sustainability. The combination of sustainability and resilience was considered in the areas of risk reduction and sustainable development for urban infrastructures, such as water distribution systems [80], built environments, and critical infrastructures [81]. Moreover, research regarding contemporary urban planning and design and the provision of energy to building structures during earthquakes was also conducted for urban risk reduction and sustainable development [65]. As for vulnerability, in the 1990s, scholars found that after disasters, the discussion on “vulnerability” was not sufficient to describe a system facing risk events, while the ability of the system to recover rapidly showed high importance. Then, “resilience” was gradually utilized in disaster research [49]. For instance, strengthening local food systems will reduce the vulnerability of the community following extreme weather events and strengthen the resilience of large chain supermarkets [82].

### 4.3. Phase III: Rapid Expansion Phase (2016–January 2021)

Articles during 2016 to January 2021 show a sharp growth trend in the number of publications compared with 2008–2015, and in total 258 articles were published.

#### 4.3.1. Timeline Visualization Analysis

In the last five years, urban disaster resilience research has attracted more and more attention from scholars, and the total number of articles has reached 258. New keywords such as “natural disaster”, “urban resilience”, and “natural hazard” have occurred and have shown a high rate of occurrence during this period. Obviously, compared with Phase II, the keywords have been constantly changing and become more diverse over time. Therefore, scholars begin to care more about how to propose effective methods to promote urban resilience to natural disasters and further apply them to practical problems, such as earthquakes and floods, etc.

#### 4.3.2. Research Category Analysis

In Figure 6, the relationship between node links displays that the keywords that emerged in the first two phases were still active at this stage. Papers that derived from high citation and top-ranked journals were analyzed in depth as follows.

(1) Disaster Risk Reduction

For disaster risk reduction, in total there are 103 articles are in this category, and they could be classified into three sub-categories, which are research in different disaster phases, relationships between disaster and resilience, and exploration in resilience enhancing strategies.

(1.1) Research in different disaster phases

In this sub-category, three disaster phases are included, which are pre-disaster, emergency rescue, and post-disaster.

In the pre-disaster phase, emergency evacuation planning and disaster recovery planning were focused on improving urban resilience [83]. In flood risk mitigation, urban spatial planning and flood risk maps were considered to be useful methods when conducting urban planning activities [84]. The emergency shelter planning was focused on enhancing urban disaster resilience, and a forecasting method to estimate the time-varying shelter demand was proposed [85]. Resilience in the context of evacuation planning with street capacity considerations was introduced [86]. Moreover, social capital was considered to cultivate disaster resilience during disaster preparedness [87].

Secondly, community resilience in the emergency context was discussed. Community resilience is principally recognized as the product of risk reduction, emergency response, and post-disaster interventions [88]. The Queensland Emergency Services Cadets Program aims to build resilience in communities by providing opportunities to young people in training skills and promoting confidence and a sense of purpose in emergency rescue [89]. Leadership is a key element during emergencies and the effective information provided by municipal authorities should meet the victims’ needs [90]. Two worldwide programs called “Earthquake and Safet” and “Safe Schools-Resilient Communities” were formulated by the International Institute of Earthquake Engineering and Seismology (IIEES) in Iran to increase preparedness, promote public participation, and develop emergency response [91].

Thirdly, more attention to community resilience was paid in the post-disaster phase, including the resilient social processes [92], the role of social capital, personal networks, and emergency responders [93]. A consistent significant positive correlation was presented between resilience and cohesion, and the mean intensities of these two features show place-specific differentiation [52,94]. In addition, after major flooding disasters, the post-disaster resilience was highlighted through expanding local behavioral health service delivery capacity [95], and after the Great East Japan Earthquake and Tsunami in March 2011, the co-evolutionary dynamics between perceptions of community resilience and the formation of supportive links among residents were explored [96].

(1.2) Relationships between disaster and resilience

Disaster resilience is a complex problem and needs truly interdisciplinary research [97]. The goal of urban resilience is to shape a “culture of resilience” to mitigating damage and loss from disasters to the maximum degree [83]. Urban risk management in Africa was discussed, and the rise of resilience paradigms in urban development offered useful ways for risk-sensitive urban development [98]. The extent that community resilience mitigates climate-related damage was evaluated by a composite index in Fiji based on survey data [99]. The urban sectors were denoted that have better disaster governance for determining community resilience than rural sectors in three aspects, which are the responsibility of multiple levels of governments, the resource supply availability after disasters, and the politics of disaster [100,101]. The disaster entrepreneurship is the private sector and takes advantage of business opportunities and serves community stakeholders to create value during or after a disaster [102]. A local community-driven bottom-top approach to mitigate risk and disseminate appropriate disaster risk information was proposed to promote community-based resilience [96,103]. A conceptual model of a coupled human-landscape system in Swiss Alpine communities was developed to assess risk and community resilience, which is a theoretical innovation in developing disaster risk management plans for communities [104]. In practice, the disaster resilience practices in residential communities were identified, and the stakeholder views on disaster resilience practices of residential communities were analyzed [105]. Additionally, seven recommendations for enhancing the integration of natural hazard science into disaster risk reduction were set out, which can better contribute to the planning and development of sustainable and resilient communities [106].

(1.3) Exploration in resilience enhancing strategies

Both researchers and practitioner groups agree that it is necessary to maximize community resilience benefits. The community skill requirements for enhancing disaster resilience were considered to relate to building environment professionals and five disaster resilience dimensions, which include society, economy, technology, environment, and institutions [107]. Several resilience enhancement strategies, including innovative models and methods, have been proposed. For example, a novel methodology based on a resilience metric called the Climatic Hazard Resilience Indicators for Localities (CHRIL) was established, using a fuzzy multi-criteria decision analysis with a participatory geographic information system approach, which can encourage stakeholder participation and communication between planners in shaping metropolitan land-use policies [108]. A comprehensive approach, combining empirical experience, simulative methods, and engineering, was proposed, which could determine the quantities needed for urban areas’ resilience and risk assessments and further help create more resilient cities [109]. The Community Disaster Resilience Scorecard self-testing was recognized as a useful method for improving community disaster resilience. Self-testing revealed that cross-community cooperation, better communication, and maximizing opportunities to compare plans are necessary for strengthening disaster resilience [110]. Sustainable flood memory, lay knowledge, and the development of community resilience from the theoretical and practical perspective were explored in relation to future flood risk [111]. Moreover, virtual communities of practice (VCoPs) were adopted for improving organization resilience, and a Delphi study was conducted to evaluate the contribution [112].

Resilience enhancement strategies have also been explored from the perspective of infrastructure. A spatial data infrastructure was designed to access geospatial data, which could be used to analyze disaster emergencies and support decision makers in mitigating disaster risks and enhancing community resilience [113]. Additionally, a seven-layer classification of infrastructure to improve community disaster resilience was proposed, which facilitates an understanding of the interdependencies within the layers and an analysis of various communities’ needs in post-disaster recovery [114]. Moreover, a sequential discrete optimization approach was proposed as a decision-making framework at the community level for post-hazard recovery. The proposed methodology overcomes the limitations of dimensionality and manages large-scale infrastructure systems following disasters to enhance community disaster resilience [115].

(2) Specific Disaster Resilience Research

For specific disaster resilience research, there are 90 articles in total in this category. Most studies focus on natural disaster resilience research. Additionally, community resilience building and strengthening has also been explored for human-induced disasters, such as oil spills, environmental disasters [116], and terrorist disasters [117], etc.

Large numbers of people who live in urban areas are increasingly exposed to hydrological risks, weather risks, and sea-related risks [118,119,120]. Flood disaster is one kind of natural disaster that has been focused on in many urban and community resilience studies. Resilient flood risk management and resilience strengthening strategies of urban retailers have been examined for coastal cities and riverbank areas, including Hong Kong, Singapore, and Kaohsiung [121,122,123]. In the wetland communities that suffer from flash flood disasters, considerations should be given to education development, a diverse range of skills, and social perception among the local population to enhance the community’s post-disaster adaptive capacity [124,125]. An urban flood resilience assessment method, a modified Drivers–Pressures–State–Impact–Response framework, was proposed for improving the flood resilience of cities [126]. In addition, Vamvakeridou-Lyroudia et al. (2020) proposed an integrated and participatory methodological approach to enhance the resilience of interconnected critical infrastructures to urban flooding [127].

Moreover, other natural disasters, such as tornadoes, hurricanes, and seismic disasters, have also been the focus of resilience research. The post-disaster recovery of the Illinois tornadoes was examined, which advances the theorization of disaster communication ecology and fosters community resilience [35]. The relationship between communities’ risk perceptions and resilience was investigated, and the results showed that community capital and economic resilience could strengthen perceptions of hurricane risks [128]. A quantitative framework to model recovery patterns of economic activity was introduced and applied in a retrospective study of Hurricane Katrina, which provides actionable information for prompting resilience in diverse communities and different phases of a disaster [129]. Additionally, the resilient post-earthquake recovery was also discussed, including critical factors in contributing to resilient recovery [130], the role of institutional initiatives and the communities’ response to earthquake disaster [131], the relevance of earthquake-stricken resilience and disaster risk reduction efforts [132], and the short- and long-term societal impact of prolonged power outages caused by disasters [133].

(3) Resilience Assessment

For resilience assessment research, there are 48 articles in this category. Most studies focus on the measurement of community resilience. Various resilience assessment research was conducted and covered multiple aspects of resilience. For instance, using a questionnaire survey and a subjective assessment method, a flood resilience indicator system was established to measure community resilience in the Khyber Pukhthunkhwa province of Pakistan [134]. Similarly, a set of indicators in terms of social, economic, human, institutional, and environmental aspects were constructed to measure community disaster resilience and were applied to 229 local municipalities in Korea [135]. For coastal communities, a framework that innovatively disaggregates hurricane community resilience to the individual level and quantifies resilience for the individual residential buildings was proposed [136]. Moreover, for the coastal areas of China, the main driving factors of overall community resilience were identified, including a robust and developed economic system, effective education, training programs, and adequate investment [137]. The resilience inference measurement (RIM) model was used for assessing the community resilience to drought hazards of all 503 counties, and the results showed that the social, economic, agriculture, and health sectors were identified as the main resilience indicators [138].

Moreover, some resilience assessment methods were also developed. The Resilience Performance Scorecard was used to measure the community/urban disaster resilience of Australia and Lalitpur, Nepal, which is a multilevel and multi-scale self-evaluation tool that empowers stakeholders to assess resilience parameters [139,140]. Multilevel indicators in risk and resilience monitoring were developed by using Principal Component Analysis and Varimax Factor Analysis, and the structure of the indicators provides guidance on how to adjust risk management for different scales [141]. To assess the landslides-oriented urban disaster resilience, a Delphi-Analytic Hierarchy Process (Delphi-AHP) model and the Support Vector Machine (SVM) were applied [142].

(4) Combination Research

For combination research, two important concepts, sustainability and vulnerability, were continuously focused on in disaster resilience research. According to a human-centric and social perspective, an integrated framework was proposed to quantify disaster resilience of the urban system. The sustainability of the urban system was considered in the resilience assessment, as both resilience and sustainability could reduce risk and supply resources to post-disaster recovery [26,27]. Resilience was also proposed as a foundation for sustainability in social sustainability-oriented tourism concepts and resilience planning [28]. An integrated framework for multisector infrastructure asset management to make cities more sustainable and resilient against increasing threats was established [23].

In the combined research with vulnerability, resilience has emphasized that given the relationship with vulnerability, both could share similar factors, such as disaster mitigation strategies [143]. Using exposure, damage, and recovery indicators, the relationship between vulnerability and adaptability was denoted through the resilience inference measurement (RIM) model, which aimed at quantifying resilience to climate-related hazards [144]. The term vulnerability was considered strongly linked with resilience and also conceptualized as the opposite of vulnerability for the ability to resist impacts of disasters [118]. A vulnerability-resilience indicator multi-criteria analysis was used to show the variability and contribution rate of the water-related risks [145]. The Spatial Multi-Criteria Evaluation (SMCE) was applied to assess the vulnerability-resilience of at-risk communities at Kelud Volcano [143]. In practice, the Vulnerability to Resilience (V2R) program implemented in Bangladesh showed that sustainable livelihoods had been well connected with disaster risk reduction [146]. Towards improving the resilience of cities, the problem of road maintenance/development was formulated as a mathematical model, which reduced the vulnerability to disruption [147].

### 4.4. Features of the Four Research Categories

According to the analysis of the four research categories in each phase, the research features of each category can be further summarized systematically, as shown in Table 6.

## 5. Discussion

**Figure 7:**
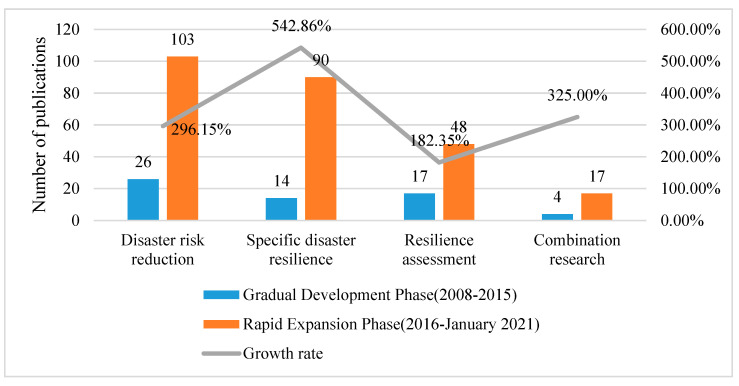
The development of the publications in four categories from Phase II to Phase III.

The number and growth rate of publications from Phase II (2008–2015) to Phase III (2016–January 2021) are shown in Figure 7.

### 5.1. The Major Research Categories

Figure 7 shows that disaster risk reduction and specific disaster resilience research are two major research categories, and the number of published articles is significantly higher than the other two categories. Nowadays, cities worldwide are vulnerable to severe influence from a range of stresses and shocks that come from both natural disasters and human-made disasters. Resilience is the ability of the urban system to maintain continuity through all stresses and shocks while positively adapting and restoring towards sustainability. Thus, the research focusing on disaster risk reduction and specific disaster resilience dominates the major proportion.

According to the above literature review, the research in the category of disaster risk reduction covers many aspects, such as relationships between disaster and resilience, the impacts of disasters, etc. The main aim of the research is to establish disaster risk reduction measures and resilience enhancing strategies, such as a variety of practical applications and promotion activities aiming to enhance urban/community disaster resilience. Moreover, research in the category of specific disaster resilience research focuses on some specific disasters, including natural disasters and human-made disasters. Based on the above review, it can be seen that natural disasters, especially floods, earthquakes, tsunamis, and cyclones, are the main kinds of disasters that are focused on. The adaptation and recovery conditions for the detailed disasters were discussed. Additionally, some topics, such as social capital, community education, and risk perceptions, etc., were also explored in some specific disaster resilience research. In addition, the number of publications in the field of resilience assessment research has seen a significant increase during Phase III, which displays the high potential research prospect. Discussions on resilience assessment research refer to a variety of methods, such as frameworks, models, indicator/index systems, and factors, etc. In the meantime, case studies are usually used to prove the value of theoretical resilience assessment methods in practical applications.

### 5.2. The Research Categories with High Growth Rate

According to Figure 7, the two categories "specific disaster resilience research” and “combination research” present a much higher growth rate of publications from Phase II to Phase III, both of which are higher than 300.00%. The category “specific disaster resilience” displays the highest growth rate at 542.86%. In this category, studies focus on specific disasters, including natural disasters and human-made disasters. Based on the above analysis and realistic background, it can be concluded that natural disaster resilience is a promising research direction and shows important research significance, that is, effective disaster response and maximization of loss reduction. The relevant content involves the establishment of resilience models and the improvement of restorative capacity, etc. In the meantime, great attention has also been drawn to specific disasters or problems, such as public health, flood risk, tsunami, climate change, public–private partnerships, and women’s empowerment. Additionally, the topic of human-induced disaster possesses a definite proportion in the field of community resilience research.

The growth rate of articles in the category “combination research” is 325.00%. In this category, sustainability and vulnerability are the two main concepts that were integrated into the urban disaster resilience research. The UN-Habitat indicated that all cities are vulnerable to the shocks of disasters, including natural disasters and human-made disasters, and resilience is considered in the global agendas as a crucial concept to adapt to the disasters and transform towards sustainability [148]. In addition, vulnerability was considered a key variable in disaster risk reduction, which is strongly linked with resilience and the opposite of resilience. The relationship and assessment index of vulnerability and resilience were also explored. Sustainability was the aim of resilience research, especially in the post-disaster recovery phases. Additionally, resilience has emphasized the necessity for urban sustainability. Three generalized frameworks for organizing resilience and sustainability could be concluded: (1) sustainability as a component of resilience, (2) resilience as a component of sustainability, and (3) resilience and sustainability as separate objectives [149]. Relevant research includes the sustainability-oriented resilience planning, sustainability integration frameworks in resilience assessment, etc.

### 5.3. The Research Corresponding to Practices

The UN-Habitat, the World Bank, the Rockefeller Foundation, and many institutions launched a number of programmes to further the practice, knowledge, and awareness of urban resilience. The partnerships of all the major actors working in urban resilience were forged, the aim of which is to bring more understanding and cohesion around urban resilience thinking, in particular to local governments. The existing research corresponds to the urban resilience practices in the aspects of timeline and contents.

#### 5.3.1. Timeline

In 2005, the Second UN World Conference on Disaster Reduction was held in Kobe, Japan. A document, the Hyogo Framework for Action 2005–2015: Building the Resilience of Nations and Communities, was deliberated and approved. This framework defined strategic goals and initiative actions of disaster risk reduction from 2005 to 2015. Moreover, the programme “Making Cities Resilient: My city is getting ready!” was launched by the UN-Habitat in May 2010 to address urban risk and resilience and local risk governance. The campaign was assured through two documents related to the Sendai Framework for Disaster Risk Reduction issued in 2015 and 2016, respectively. The programme created a broad platform and alliance for dialogue and developed some basic tools for resilient capacity development and implementation. The programme will continue beyond 2020 with the recommendations of participants and partners. Moreover, the programme “100 Resilient Cities” was created by the 100 resilient cities in 2013 and focused on natural disasters and human disasters. The Medellin Collaboration for Urban Resilience was launched in 2014, which gathered the most prominent actors committed to building resilience globally, including UNISDR, Rockefeller Foundation, The World Bank Group, 100 Resilient Cities, etc. In addition, the Global Alliance for Urban Crises was established in May 2016. The leaders and members of the alliance have the resources and capacities to mitigate risk impacts on vulnerable conditions.

Except for the projects launched by various international alliances and non-governmental organizations, some cosmopolitan cities, such as New York, London, Chicago, Rotterdam, and Tokyo, etc., have also joined the ranks of the development plan for enhancing urban resilience. Specifically, the 2015 plan for New York City, “One New York: The Plan for a Strong and Just City (OneNYC)” was originally released in 2007 and again in 2011 under the name “PlaNYC”. This plan presented a grand vision of building a growing, prosperous, fair and equitable, sustainable, and resilient city. Additionally, Tokyo is located in the volcanic earthquake zone around the Pacific Ocean, and natural disasters such as earthquakes and tsunamis occur frequently. In December 2014, the strategic report, “Creating the Future: The Long-Term Vision for Tokyo”, was released by the Tokyo Metropolitan Government. The purpose of the report is to build into a world-class metropolis. In addition, the London Plan was launched by the UK government in March 2015, which aimed at building to be a top global city by 2036.

According to the statistics above, the programme regarding urban resilience launched by the UN-Habitat started in 2010 and implemented the main contents from around 2015. The above review demonstrates that the existing research progressively increased from 2007 to 2014, while from 2015 to date, it presents a rapid expansion. Thus, the growth trend of the published articles in the gradual development phase and rapid expansion phase corresponds with the programme practices in the aspect of the timeline.

#### 5.3.2. Contents

The RESCUE project, “Resilience to Cope with Climate Change in Urban Areas”, focuses on the water system and aims to provide practical tools to end-users facing climate change challenges and building more resilient cities. The current and future climate change scenarios and other hazards were considered in this project. The Risk Nexus Initiative is a partnership formed by the leading institutions, which is engaged in resilience, sustainability, and risk management, with rich risk management experience. The central focus of the initiative is to ensure that all new development is fully risk-informed, including climate change adaptation, disaster risk reduction, early warning, cities, and urban development, etc. Moreover, the Post-Disaster Housing project was proposed by the UN-Habitat, and the aim is to improve the quality of reconstructed houses. This project focus on the disasters such as floods, earthquakes, etc. and the restorative capacity of the urban system. Similar contents, such as earthquakes, fires, floods, are also focused on by the 100 Resilient Cities, which was created by the Rockefeller Foundation in 2013. As for the World Conference of Disaster Risk Reduction, different strategic goals have been established by the United Nations International Strategy for Disaster Reduction based on different era backgrounds. The Hyogo Framework for Action 2005–2015 determined a more comprehensive system for the planning, implementation, and assessment of disaster risk reduction activities. In 2015, a global disaster prevention and mitigation goal with specific projects and deadlines was firstly proposed in the Sendai Framework for Disaster Risk Reduction 2015–2030. This framework identified seven global targets and four priority areas, which involved broadening the scope of disaster risk reduction significantly and reducing the number of people affected by global disasters and direct economic losses, etc.

Along with the frequent occurrence of urban crises, such as climate change and seismic disasters, etc., enhancing urban resilience has increasingly become the strategic choice to cope with sudden shocks. More and more cities began to attach importance to the construction and development of resilient cities. A series of urban development plans were designed to improve urban resilience. For instance, the official document, “Creating the Future: The Long-Term Vision for Tokyo”, refers to four aspects of resilient city construction measures, including infrastructure resilience, economic resilience, institution resilience, and social resilience. In 2007, the New York City government was devoted to addressing New York City’s economic growth, sustainability, and resilience, etc. Moreover, strategies for addressing income inequality and plans for managing climate change impacts were laid out, while the establishment of a platform for another century of economic growth and vitality was considered. Similarly, the London Plan also set out an economic, environmental, transport, and social framework to deal with a variety of important city-building elements. Specifically, six detailed development visions that are associated with economic vitality, quality of life, sense of community belonging, urban environmental quality, low-carbon environmental protection, and infrastructure were proposed.

Thus, the contents that disaster risk reduction, specific risks (climate change, flood, and earthquake, etc.), and system sustainability are also the research hotspots of existing studies, which indicate the correspondence between the practices and research.

### 5.4. Future Research Directions

The two categories, specific disaster resilience research and combination research, present high growth rates and deserve deeper research. In recent years, a large number of disasters have occurred around the world and have brought some negative effects on urban development and people’s lives. Research on specific disaster resilience research could supply useful references for disaster resilience enhancement, including disaster risk management, specific disaster response, post-disaster recovery strategies, and resilience strengthening strategies, etc. Research in this aspect still has a large space due to various kinds of disasters and their uncertainties. Moreover, disasters are generally divided into three stages, which are also important research entry points. The resilience improvement strategies may vary in accordance with the different measures of the three stages. Therefore, specific disaster resilience could be further explored in future research.

Moreover, the urban resilience research combined with sustainability and vulnerability is also a valuable research direction in the future. With a high growth rate of research, it has been recognized that these two concepts present a close relationship with resilience. Rational urban development could only be achieved when it is both resilient and sustainable. The three concepts are the basic characteristics and abilities of urban systems, especially in the field of safety/risk management. Along with the high growth of research trends, the combination research deserves future endeavors.

## 6. Conclusions

Nowadays, cities and citizens are facing continuous and serious challenges as a result of rapid urbanization, which increases the population’s exposure and vulnerability to disasters, including natural disasters and human-made disasters. In order to reduce the negative impacts of disasters and provide a foundation for sustainable development, the importance of building urban disaster resilience is increasingly realized. This paper gives a systematic review and analysis of the literature on urban disaster resilience. The review is based on keyword statistics and includes two steps. Firstly, the statistics for the basic research information were conducted from three aspects, which are the publication years, journals, and keywords. Accordingly, the annual distribution and research hotspots of the publications were obtained. Then, the second step was about the deeper analysis of urban disaster resilience research from three phases and four categories. Specifically, combined with the keyword statistical analysis results and clustering results, the main content was classified into four hotspot topics: (1) disaster risk reduction, (2) specific disaster resilience research, (3) resilience assessment, and (4) combination research. The review for the articles was conducted from these four categories. The results show that the first two categories are the major research categories, while the second and fourth categories show a high growth rate of published articles. The categories with high growth rates could be directions for future research on urban disaster resilience. The review maps a full picture of existing urban disaster resilience research.

In addition, the four research categories are strongly associated with urban resilience enhancement, which can contribute to the establishment and development of a resilient city. Specifically, the category disaster risk reduction presents the characteristics of the whole life cycle of disasters. From a temporal perspective, this feature corresponds to the concept of resilience, and it aims to reduce risk and improve resilience from all stages of a disaster. Additionally, resilience studies and practices for specific disasters can be helpful for urban resilience enhancement, with a focus on frequent occurrence disasters, such as earthquakes and climate-related disasters, etc. Resilience assessment research mainly presents a wide variety of indicators, which is conducive to putting forward targeted recommendations for resilience enhancement. Meanwhile, numerous novel assessment methods (such as framework and model, etc.) can also provide some guidance for the practices of improving urban resilience. The combination research includes two important concepts, namely, vulnerability and sustainability. Studies on vulnerability are not sufficient to reflect the whole life cycle of disasters, and the term resilience plays a compensatory role in urban disaster research. Moreover, urban resilience research also promotes urban sustainable development. Therefore, the analysis of articles in four categories could be useful references for urban government officials and policy-makers in establishing urban resilience enhancement strategies.

## Figures and Tables

**Table 1:** Top 10 journal sources for urban disaster resilience research.

No.	Journal	No. of Articles
1	*International Journal of Disaster Risk Reduction*	37
2	*Natural Hazards*	22
3	*International Journal of Environmental Research and Public Health*	11
4	*Sustainability*	11
5	*Disaster Prevention and Management*	9
6	*Disaster Medicine and Public Health Preparedness*	6
7	*Disasters*	6
8	*Natural Hazards Review*	6
9	*Annals of the American Association of Geographers*	5
10	*International Journal of Disaster Risk Science*	5

**Table 2:** Centrality of the top 20 keywords in urban disaster resilience studies.

No.	Keywords	Centrality	No.	Keywords	Centrality
1	preparedness	0.26	11	earthquake	0.11
2	disaster risk reduction	0.20	12	urban resilience	0.11
3	policy	0.17	13	engagement	0.11
4	challenge	0.17	14	community resilience	0.10
5	reflection	0.16	15	natural disaster	0.10
6	perception	0.15	16	knowledge	0.10
7	social ecological system	0.14	17	perspective	0.10
8	hazard	0.13	18	resilience	0.09
9	risk reduction	0.13	19	tsunami	0.09
10	disaster	0.11	20	impact assessment	0.09

**Table 3:** Keywords that initially appeared in Phase III (frequency > 5).

Keywords	Year	Frequency	Keywords	Year	Frequency
natural hazard	2020	5	risk	2016	6
covid-19	2020	4	tsunami	2016	6
social capital	2018	9	cyclone	2016	5
urban resilience	2018	7	Bangladesh	2016	3
disaster risk management	2018	4	adaptive capacity	2016	3
natural disaster	2016	7			

**Table 4:** Keywords with a frequency not less than 5 in urban disaster resilience studies.

No.	Keywords	Frequency	No.	Keywords	Frequency
1	resilience	86	13	natural disaster	7
2	community resilience	54	14	urban resilience	7
3	disaster	31	15	risk	6
4	disaster resilience	15	16	hazard	6
5	disaster risk reduction	15	17	tsunami	6
6	climate change	15	18	flood	5
7	vulnerability	14	19	cyclone	5
8	community	13	20	community participation	5
9	social capital	10	21	natural hazard	5
10	disaster management	9	22	China	5
11	earthquake	8	23	preparedness	5
12	adaptation	8			

**Table 5:** Clustering table of the top eight keywords of urban disaster resilience articles.

Cluster ID	Size	Silhouette	Label (LLR)	Main Keywords
0	37	0.962	disaster risk reduction	resilience, disaster risk reduction, disaster management
1	36	0.848	community resilience	community resilience, social capital, community participation, China
2	33	0.890	adaptation	disaster, community, adaptation, risk, hazard, preparedness
3	28	0.877	disturbance	climate change
4	19	0.899	disaster resilience	disaster resilience, urban resilience, flood
5	19	0.844	earthquake	earthquake, natural disaster, natural hazard
6	17	0.937	communication	tsunami
7	16	0.924	vulnerability	vulnerability, cyclone

**Table 6:** Main features of four research categories.

Category	Research Features
Disaster risk reduction	(1) Theoretical analysis and practical applications research have been conducted for disaster risk reduction and enhancing community disaster resilience. (2) Some promotion strategies (such as critical infrastructures disaster mitigation strategies, urban governance, and urban planning, etc.) were studied to reduce disaster risk and enhance urban disaster resilience. (3) In the pre-disaster phase, emergency evacuation planning and disaster recovery planning were focused to mitigate risk and improve urban resilience. (4) More attention to community resilience was paid in the post-disaster phase.
Specific disaster resilience	(1) Research has been conducted from angles of multiple specific disasters or problems. (2) Natural disaster resilience is the main topic in this category. Flood disaster is one kind of natural disaster that has been focused on in many urban and community resilience studies. Moreover, disasters such as tornadoes, hurricanes, and seismic disasters have also been focused on resilience research. (3) Community resilience building and strengthening were also explored for human-induced disasters, such as oil spills, environmental disasters, and terrorist disasters, etc.
Resilience assessment	(1) Combined with mathematical parameters or methods, a variety of novel resilience assessment approaches, such as frameworks, models, indicators, and index systems, etc. were proposed. (2) The assessment or measurement of community resilience is a hotspot direction.
Combination research	(1) The combination of resilience and sustainability was widely applied to urban infrastructure system research. (2) Since 2016, the concepts of vulnerability and sustainability were continuously focused on in disaster resilience research. (3) Urban sustainable development was regarded as the target to conduct urban resilience research. (4) The relationship of resilience and vulnerability has been emphasized, as both could share similar factors, such as disaster mitigation strategies.

## Data Availability

Data sharing not applicable.

## Appendix A

**Table A1:** Reference classification based on three Phases.

Phase	References
Phase I (2001–2007)	Paton et al. (2001) [44], Godschalk (2003) [45], Chang and Shinozuka (2004) [46], Levy and Gopalakrishnan (2005) [47], Murphy (2007) [48].
Phase II (2008–2015)	Cutter et al. (2008) [49], Boyle (2012) [50], Januaryg and Wang (2009) [51], Townshend et al. (2015) [52], Kelman (2008) [53], Alshehri et al. (2015) [54], Wamsler (2008) [55], Stevens et al. (2010) [56], McCarthy et al. (2011) [57], Dufty (2012) [58], Xiao and Zandt (2012) [59], Prashar et al. (2013) [60], Tappenden (2014) [61], Cox and Perry (2011) [62], Bouchon and Di Mauro (2012) [62], Guo (2012) [64], Ahern (2011) [65], Gunderson (2010) [66], Fannin et al. (2012) [67], Boon et al. (2012) [68], Ainuddin and Routray (2012) [69], Aryal (2014) [70], Chen et al. (2013) [71], Mebarki et al. (2012) [72], Parvin and Shaw (2011) [73], Plough et al. (2013) [74], Sugimoto et al. (2010) [75], Smith et al. (2008) [76], Chen et al. (2008) [77], Chen et al. (2009) [78], Joerin et al. (2012) [79], Blackmore and Plant (2008) [80], Coaffee (2008) [81], Singh-Peterson and Lawrence (2015) [82], Zhai et al. (2015) [83], Davidson (2015) [97].
Phase III (2016–January 2021)	Şenol Balaban (2016) [84], Zhao et al. (2017) [85], Kimms and Maiwald (2018) [86], Straub et al. (2020) [87], Paschen and Beilin (2017) [88], Baisden (2017) [89], Cohen et al. (2017) [90], Hosseini and Izadkhah (2020) [91], Imperiale and Vanclay (2016) [92], Sadri et al. (2018) [93], Ludin et al. (2019) [94], Keegan et al. (2018) [95], Lim and Nakazato (2019) [96], Fraser et al. (2017) [98], Gawith et al. (2016) [99], Villagra and Quintana (2017) [100], Drennan (2018) [101], Linnenluecke and McKnight (2017) [102], Aka et al. (2017) [103], Hossain et al. (2020) [104], Taeby and Zhang (2018) [105], Gill et al. (2021) [106], Perera et al. (2017) [107], Hung et al. (2016) [108], Fischer et al. (2016) [109], Ludin and Arbon (2017) [110], McEwen et al. (2017) [111], Gimenez et al. (2017) [112], Sterlacchini et al. (2018) [113], Choi et al. (2019) [114], Nozhati et al. (2019) [115], Reams et al. (2017) [116], Miller et al. (2017) [117], Rey et al. (2017) [118], Heinzlef et al. (2020) [119], Crosweller and Tschakert (2020) [120], Chan et al. (2018) [121], Ling and Chiang (2018) [122], Raskin and Wang (2017) [123], Choudhury and Haque (2016) [124], Bodoque et al. (2016) [125], Hammond et al. (2018) [126], Vamvakeridou-Lyroudia et al. (2020) [127], Shao et al. (2018) [128], Qiang et al. (2020) [129], Mishra et al. (2017) [130], Ray (2017) [131], Cui et al. (2018) [132], Moreno and Shaw (2019) [133], Qasim et al. (2016) [134], Yoon et al. (2016) [135], Dong et al. (2017) [136], Qin et al. (2017) [137], Mihunov et al. (2018) [138], Arbon et al. (2016) [139], Khazai et al. (2018) [140], González et al. (2018) [141], Zhang et al. (2019) [142], Hizbaron et al. (2018) [143], Lam et al. (2016) [144], Aroua (2016) [145], Ahmed et al. (2016) [146], Mera and Chandra (2020) [147], Marchese et al. (2018) [149].
